# Case report: High-resolution, intra-operative µDoppler-imaging of spinal cord hemangioblastoma

**DOI:** 10.3389/fsurg.2023.1153605

**Published:** 2023-06-05

**Authors:** Sadaf Soloukey, Luuk Verhoef, Bastian S. Generowicz, Chris I. De Zeeuw, Sebastiaan K. E. Koekkoek, Arnaud J. P. E. Vincent, Clemens M. F. Dirven, Biswadjiet S. Harhangi, Pieter Kruizinga

**Affiliations:** ^1^ Department of Neuroscience, Erasmus MC, Rotterdam, Netherlands; ^2^ Department of Neurosurgery, Erasmus MC, Rotterdam, Netherlands; ^3^ Royal Dutch Academy for Arts and Sciences, Netherlands Institute for Neuroscience, Amsterdam, Netherlands; ^4^ Department of Neurosurgery, Park MC, Rotterdam, Netherlands

**Keywords:** µDoppler, hemangioblastoma, intramedullary tumor, functional ultrasound, spinal cord, case report

## Abstract

Surgical resection of spinal cord hemangioblastomas remains a challenging endeavor: the neurosurgeon’s aim to reach total tumor resections directly endangers their aim to minimize post-operative neurological deficits. The currently available tools to guide the neurosurgeon’s intra-operative decision-making consist mostly of pre-operative imaging techniques such as MRI or MRA, which cannot cater to intra-operative changes in field of view. For a while now, spinal cord surgeons have adopted ultrasound and its submodalities such as Doppler and CEUS as intra-operative techniques, given their many benefits such as real-time feedback, mobility and ease of use. However, for highly vascularized lesions such as hemangioblastomas, which contain up to capillary-level microvasculature, having access to higher-resolution intra-operative vascular imaging could potentially be highly beneficial. µDoppler-imaging is a new imaging modality especially fit for high-resolution hemodynamic imaging. Over the last decade, µDoppler-imaging has emerged as a high-resolution, contrast-free sonography-based technique which relies on High-Frame-Rate (HFR)-ultrasound and subsequent Doppler processing. In contrast to conventional millimeter-scale (Doppler) ultrasound, the µDoppler technique has a higher sensitivity to detect slow flow in the entire field-of-view which allows for unprecedented visualization of blood flow down to sub-millimeter resolution. In contrast to CEUS, µDoppler is able to image high-resolution details continuously, without being contrast bolus-dependent. Previously, our team has demonstrated the use of this technique in the context of functional brain mapping during awake brain tumor resections and surgical resections of cerebral arteriovenous malformations (AVM). However, the application of µDoppler-imaging in the context of the spinal cord has remained restricted to a handful of mostly pre-clinical animal studies. Here we describe the first application of µDoppler-imaging in the case of a patient with two thoracic spinal hemangioblastomas. We demonstrate how µDoppler is able to identify intra-operatively and with high-resolution, hemodynamic features of the lesion. In contrast to pre-operative MRA, µDoppler could identify intralesional vascular details, in real-time during the surgical procedure. Additionally, we show highly detailed post-resection images of physiological human spinal cord anatomy. Finally, we discuss the necessary future steps to push µDoppler to reach actual clinical maturity.

## 1. Introduction

Hemangioblastomas are highly vascularized, benign tumors (1), accountable for 2%–15% of all primary tumors in the spinal cord (2, 3), making them the third most common primary spinal cord tumor after astrocytoma and ependymoma (4). Histopathologically, hemangioblastomas are thought to consist of intricate vascular networks containing microvasculature, primarily at a capillary scale (5). Hemangioblastomas can either occur sporadically or as a part of von Hippel-Lindau (VHL) disease (6, 7), a multicentric disorder caused by an autosomal dominant tumor suppressor gene mutation, leading to multifocal and recurrent hemangioblastomas (8). In the majority of cases, hemangioblastomas present as intramedullary lesions, with only sporadic reports of combined intramedullary-extramedullary or exclusively intradural, extramedullary presentations of the disease (9).

To this day, surgical removal of the lesion remains the primary choice of treatment (10), with literature consistently reporting the importance of achieving total tumor resection in terms of minimizing recurrence of disease and improving functional outcome (3, 11). However, the benefit of total versus subtotal tumor resection is highly dependent on the surgical safety of the procedure and risk of iatrogenic post-operative neurological deficits (12). What is more, intra-operative identification of tumor location and radicality of resections remains challenging, especially as routine MRIs have led to an evolution of case load towards earlier detection of small tumors or even incidental findings (13). In all cases, the use of intra-operative surgical tools can be of great importance to ensure safe and complete surgical resection.

Current clinical practice for spinal cord tumor resections relies heavily on a combination of pre-operative imaging (CT/MR/MRA) combined with electrophysiological intra-operative neuro-monitoring (IONM). Although literature shows that IONM can significantly improve the prevention of neurological damage during surgery (14), there is still a considerable percentage of patients who experience significant long-term neurological deterioration (12, 15, 16), despite use of IONM. What is more, relying on pre-operative images to guide real-time intra-operative decision-making is fallible, especially in the spinal cord, where the laminectomy, myelotomy, locoregional swelling and bleeding, as well as shifts due to the resection cavity itself can significantly change the field of view as the surgery progresses, disturbing the match with pre-operatively acquired images, despite the latest neuro-navigation and -tracking software.

Intra-operatively, ultrasound and its submodalities (e.g., Doppler, Contrast Enhanced Ultrasound (CEUS)) have become a more common practice during spinal procedures given their many benefits including ease of use of the techniques, mobility, and availability of real-time feedback during surgical resection (17–27). For example, several reports in literature on CEUS demonstrate how the technique could provide new insights on localization (20, 21, 26), diagnosis (21) and surgical boundaries (20, 26) during intra-operative resection of spinal cord tumors.

Over the last decade, µDoppler-imaging has emerged as a new, high-resolution, contrast-free sonography-based technique which relies on High-Frame-Rate (HFR)-ultrasound and subsequent Doppler processing. In contrast to conventional millimeter-scale (Doppler) ultrasound (28), the µDoppler technique has a higher sensitivity to detect slow flow in the entire field-of-view which allows for unprecedented visualization of blood flow down to sub-millimeter resolution. The sensitivity to slow flow is attributed to the large amount of frames available to calculate the Doppler signal from and ability to separate it from the frame wide tissue motion (29–31). Previously, our team has demonstrated the potential of µDoppler-imaging and its functional counterpart called ‘functional Ultrasound (fUS)’ during awake brain tumor resections, where we showed highly detailed functional maps and vascular morphology of a range of low and high-grade gliomas (31). Additionally, our team has evaluated the potential of µDoppler-imaging in the context of a cerebral arteriovenous malformation (AVM) (32), where the technique was able to identify key anatomical features including draining veins, supplying arteries and microvasculature in the AVM-nidus intra-operatively.

Like many other developments in Neurosurgery, the focus of µDoppler-imaging so far has primarily been on cerebral pathology, with only a handful of animal (33–36) and in human (37, 38) studies showing the potential for spinal cord imaging, with one in-human study in particular focusing on functional images acquired within the context of Spinal Cord Stimulation (SCS) for pain treatment (39). What would make µDoppler-imaging specifically valuable for the context of spinal cord hemangioblastomas, is the technique's unique potential to provide high-resolution, real-time images of the vascular network of the lesion. Literature reports recommendations on improving surgical safety and efficacy during hemangioblastoma resection by focusing on vascular details specifically (12). Compared to currently available ultrasound-based techniques such as CEUS which aim to image these vascular details (20, 21, 26), µDoppler-imaging would be able to achieve the same if not better resolution images without the need for a contrast agent (32). This means that µDoppler-imaging is continuous in nature, whereas CEUS is contrast bolus-dependent (20, 21, 26). Similarly, compared to conventional Doppler, µDoppler-imaging is able to reach far superior resolutions (in the range of 100–500 µm, depending on the transducer frequency) (40). µDoppler-imaging might therefore be a valuable addition to provide real-time, high-resolution vascular details to guide hemodynamics-based surgical decision-making in the OR, especially when macroscopic or pre-operative identification of vasculature is not sufficient.

Here we describe the first application of µDoppler-imaging in the case of a patient with hemangioblastomas located in the thoracic spinal cord. We demonstrate how µDoppler is able to identify intra-operatively and with high-resolution, key anatomical and hemodynamic features of the lesion. In contrast to pre-operative MRA, µDoppler could identify intralesional hemodynamic details, in real-time during the surgical procedure. Additionally, we show post-resection µDoppler-images of physiological human spinal cord anatomy. Finally, we discuss the necessary future steps to push µDoppler reach actual clinical maturity.

## 2. Case description

### 2.1. Patient characteristics

**Figure 1:**
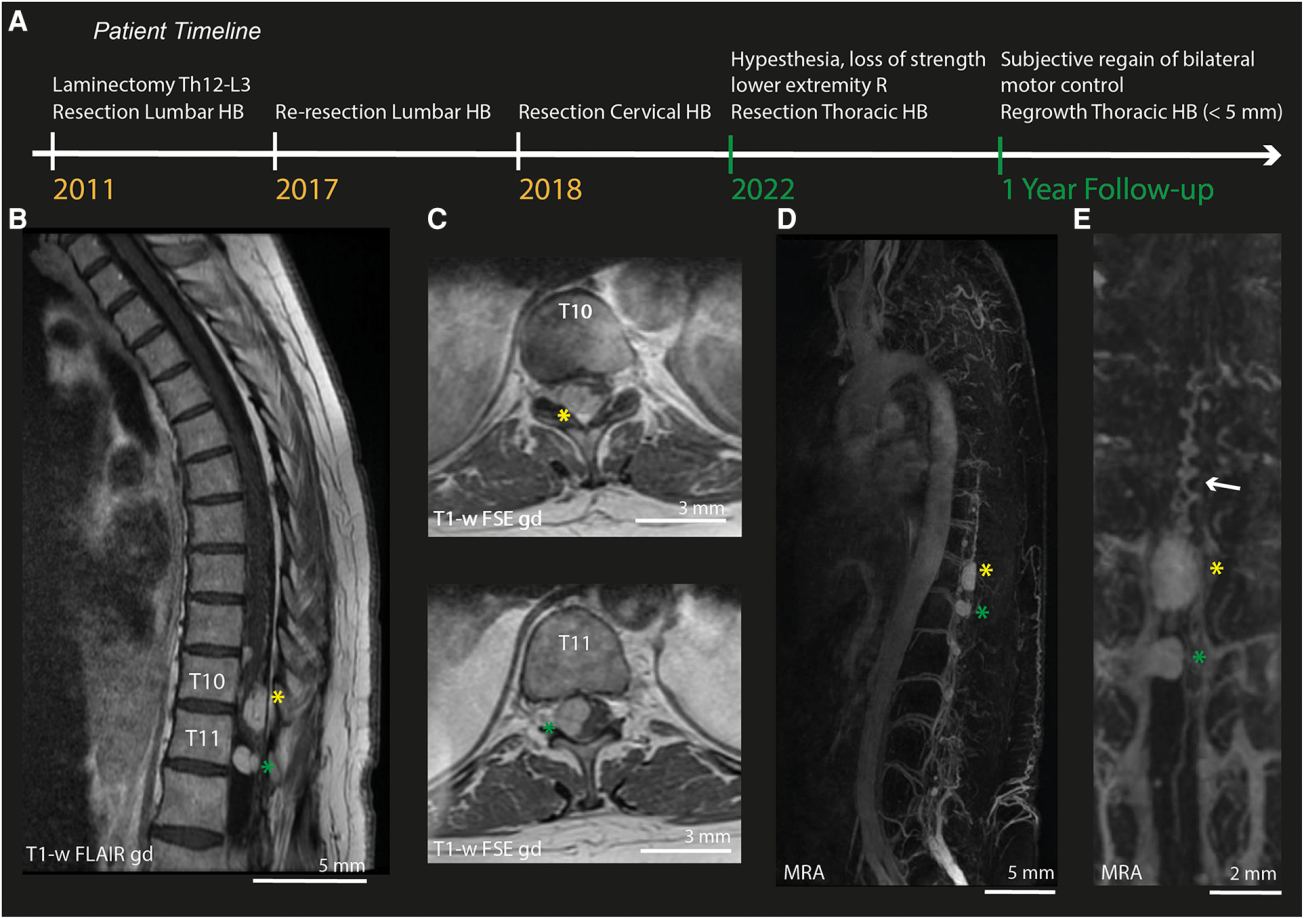
Pre-operative MRI and MRA. (**A**) Timeline of relevant surgical treatments undergone by the patients described in this case. Green labels indicate events during this episode of care. (**B**) Sagittal MRI-section of the thoracic spinal cord (T1-weighted FLAIR after gadolinium). (**C**) Axial MRI-slices (T1-weighted FSE after gadolinium) of lesion 1 at T10 (*yellow asterix*) and lesion 2 at T11 (*green asterixis)*. (**D**) MRA of the full spinal cord. (**E**) Zoom in on the MRA of both lesions. HB, Hemangioblastoma; R, Right; T1-w, T1-weighted; gd, gadolinium; MRA, Magnetic Resonance Angiography.

The patient is a female in her 60's with an extensive prior history of hypertension and recurrent spinal hemangioblastomas, for which she had three prior surgical procedures: two procedures to remove intramedullary hemangioblastomas in the lumbar region (6 years apart, including laminectomy Th12-L3) and one procedure to remove a high cervical, intramedullary hemangioblastoma (Figure 1A). This surgery was complicated by neurological deterioration. After rehabilitation, she was able to walk independently with the aid of crutches. Four years after the last surgical procedure, the patient returned with complaints of loss of strength in the right leg and shooting pains towards the foot. Neurological examination showed complete loss of right-sided lower leg strength (MRC gastrocnemius (GC) 0, Tibialis Anterior (TA) 0), and pre-existent weakness in the left leg (overall MRC 4). Additionally, the patient reported hypesthesia and loss of sharp-dull distinction on the lateral side of the right-sided lower leg and foot.

### 2.2. Pre-operative imaging

Pre-operative imaging (MRI/MRA) confirmed the presence of multiple intradural hemangioblastomas. The largest (lesion 1) appeared to be both intra- and extramedullary and was located at Th10-Th11 on the right posterior side of the myelum (1.0 cm × 1.4 cm × 2.2 cm, Figures 1B–C), causing myelum compression. An additional, extramedullary lesion was found at level Th11-Th12 (lesion 2). MRA (Figure 1D) did not show hypertrophy of the radiculary artery or dural fistuling. Prominent, probably venous vessels were seen directly caudal from lesion 1, suspected to be formed after local hemodynamic changes due to compression and/or congestion (Figure 1E).

### 2.3. Ethical statement

The patient was treated at the Department of Neurosurgery of Erasmus MC in Rotterdam. Prior to inclusion, written informed consent was obtained in line with the National Medical-Ethical Regulations (MEC2020-0440, NL67965.078.18).

## 3. Imaging procedure

### 3.1. MicroDoppler data acquisition

High-frame-rate (HFR)-acquisitions were performed using our experimental research system (Vantage-256, Verasonics, United States) interfaced with a L8-18I-D linear array (GE, 7.8 MHz, 0.15 mm pitch, probe footprint of 11 by 25 mm) or a 9l-D linear array (GE, 5.3 MHz, 0.23 mm pitch, probe footprint of 14 by 53 mm). Acoustic safety measurements were performed in collaboration with our department of Medical Technology prior to obtaining medical ethical approval to perform this study. For all scans we acquired continuous angled plane wave acquisition (10–12 angles equally spaced between −12 and 12 degrees) with a PRF ranging from 667 to 800 Hz depending on the imaging depth and transducer. The average ensemble size (number of frames used to compute one Power Doppler Image (PDI)) was set at 200 angle-compounded frames from which the live PDIs were computed, providing a live Doppler FR ranging between 3 and 4 Hz. The PDIs as well as the raw, angle compounded beamformed frames were stored to a fast PCIe SSD hard disk for offline processing purposes. Parallel to our HFR-acquisitions, patient's vital signs (EKG, arterial blood pressure) were recorded using a National Instruments’ *CompactDAQ* module (NI 9250) at 500 Hz and stored for post-processing purposes.

**Figure 2:**
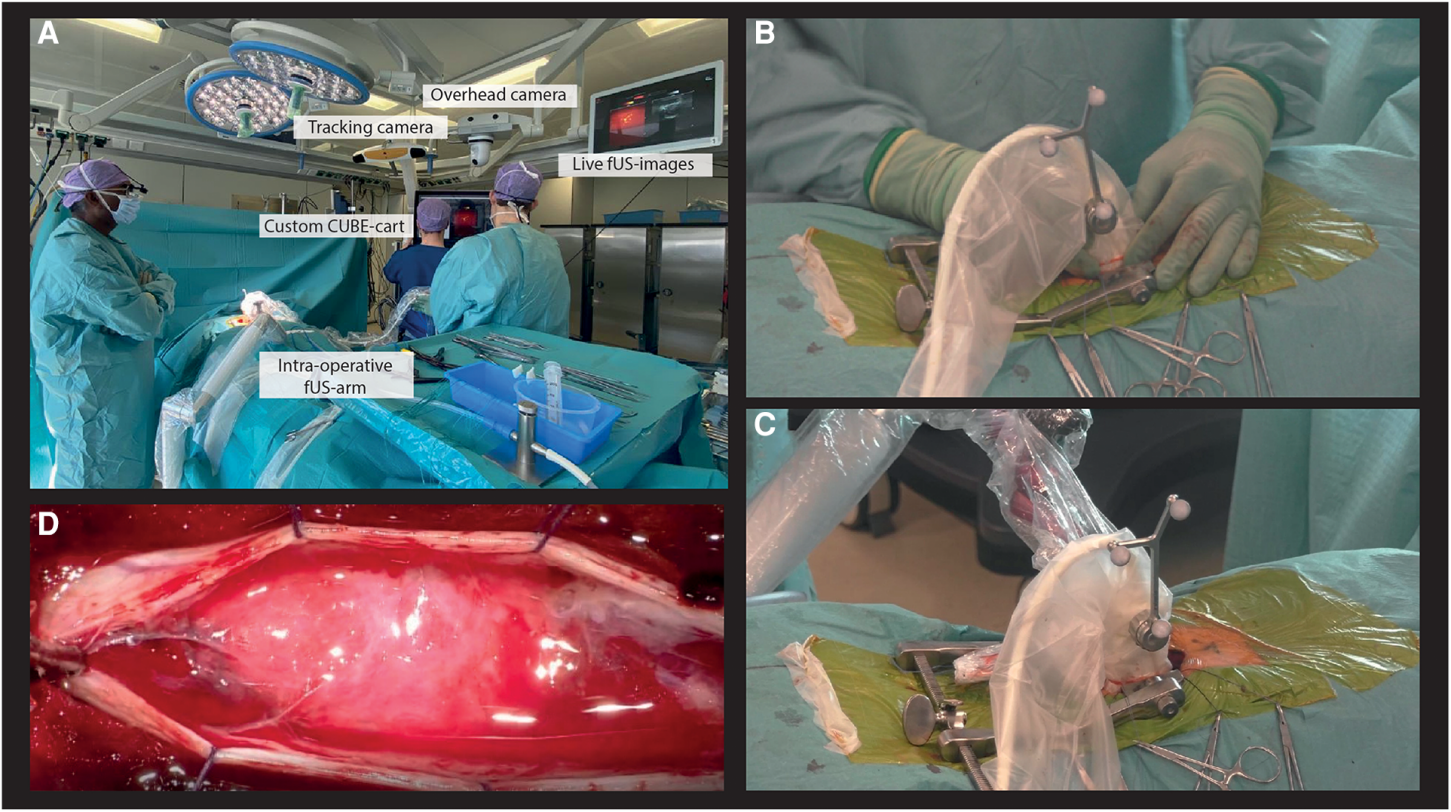
Intra-operative experimental acquisition of µDoppler-images. (**A**) Our custom CUBE-cart integrated into the conventional neurosurgical OR. (**B**) Example of a hand-held acquisition along a continuous trajectory. (**C**) Our intra-operative surgical arm used to stabilize the probe of ROI. (**D**) Microscopic view of the spinal cord after opening the dura.

To make our PDIs trackable in the OR, we integrated our transducers into Brainlab neuronavigation software by attaching the conventional optical tracking geometry to the transducer casing using custom-made 3D-printed attachments. An overhead camera recorded the surgical field as the surgeon performed µDoppler-acquisitions and removed the tumor. Through integration of our custom CUBE-cart in the OR-system, our live PDIs were displayed in real-time on the OR-screens (Figure 2A).

### 3.2. Intra-operative imaging procedure

Our experimental image acquisitions were integrated into the conventional surgical workflow, with an acquisition session both pre- and post-resection. First, the patient was placed in prone position and head-fixated in the Mayfield. A medial incision was made at the level of T9-T11, before stripping paraspinal muscles from the spinous processes and inserting a wound distractor. A laminectomy was performed from T9-T11, revealing the dura surrounding the spinal cord. The pre-resection HFR-acquisitions were performed prior to durotomy. First, hand-held 2D-images were made along a continuous trajectory spanning the full axial and sagittal length of the exposed myelum (Figure 2B) for orientation purposes. Next, stable acquisitions of 30 s were made by placing the probe over a ROI using a modified intra-operative surgical arm (*Trimano, Gettinge*) with a transducer-holder (Figure 2C). In sagittal plane, the surgical field allowed for positioning of both the L8-18I-D linear array and 9l-D linear array. For the axial plane, the surgical field was too narrow for the larger 9l-D array, so only the L8-18I-D linear array could be used in this context. Saline was added frequently to the operating field by the OR nurse to ensure adequate acoustic coupling during imaging. After the first HFR-acquisitions, the dura was opened (Figure 2D). Both tumors were removed microscopically and under guidance of IONM including measurements of Somatosensory Evoked Potentials (SSEPs) and Motor Evoked Potentials (MEPs). The most cranial lesion (*lesion 1*) revealed to have both an intramedullary and extramedullary component. The caudal lesion (*lesion 2*) revealed to be only extramedullary. Finally, the post-resection µDoppler-acquisitions were performed, again both hand-held and using the intra-operative surgical arm. The total intra-operative acquisition time of the µDoppler-data was around 30 min.

### 3.3. MicroDoppler data processing

In offline processing, PDIs were computed using an adaptive SVD clutter filter (*20% cut-off percentage*) over each ensemble and mapped onto a 100 µm grid using zero-padding in the frequency domain. The ensemble size was kept similar to the one used in acquisition (ne = 200). Given the significant, mostly in-plane motion due to the patient's breathing, single PDIs at the end of the inhale or exhale were manually selected from each dataset to ensure presentation of the most stable images.

Color Doppler Images (CDIs) were computed by taking the mean of the difference of the instantaneous phase signal for all frames in one ensemble as described by Kasai et al. (41). All initial µDoppler-data processing was performed using custom scripts in Matlab 2020b (MathWorks, Inc.).

## 4. Results

### 4.1. Pre-resection μDoppler images

**Figure 3:**
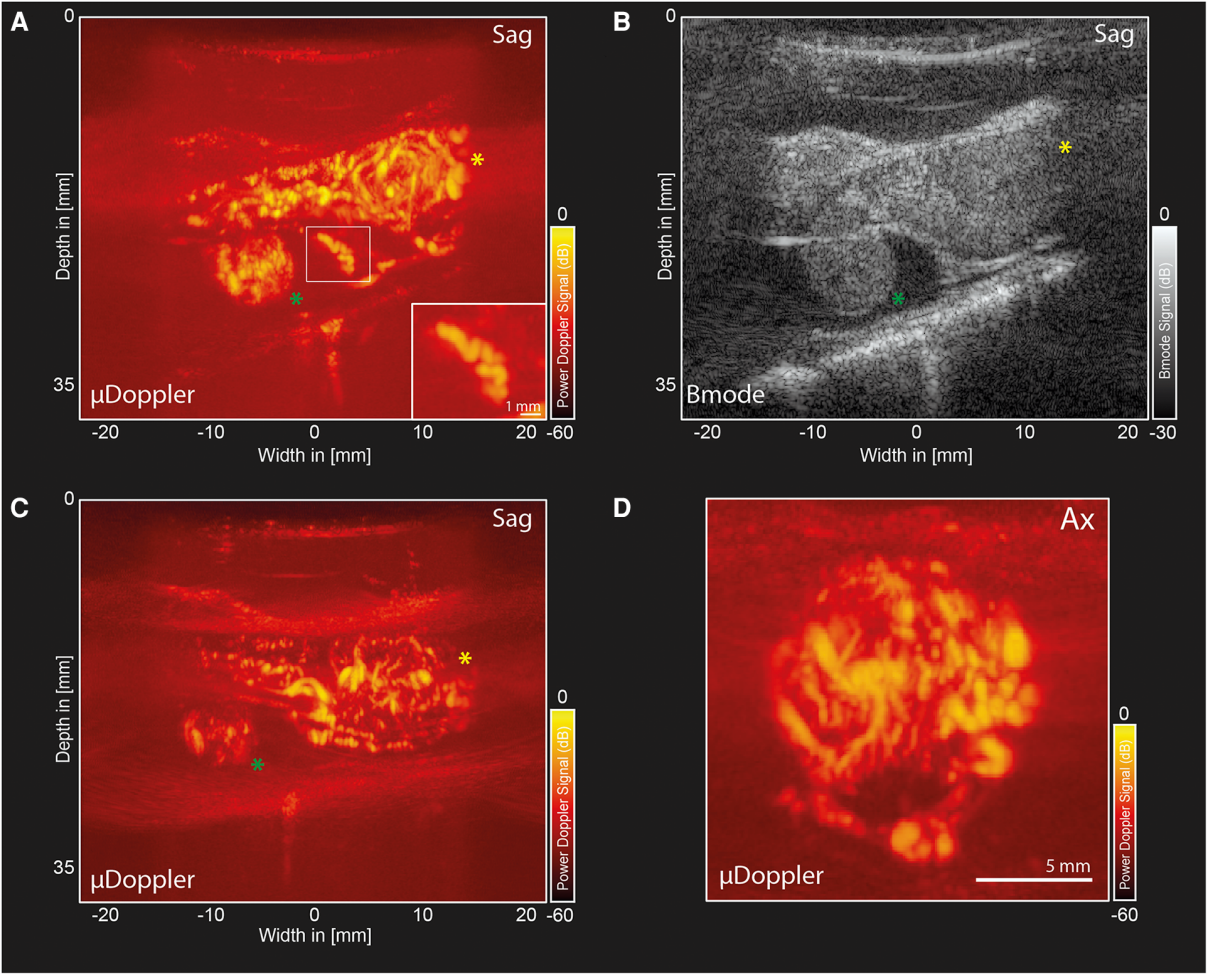
µDoppler-images of two hemangioblastoma foci. (**A**) Sagittal µDoppler-image spanning both hemangioblastoma foci. Lesion 1 (*yellow asterix*) and Lesion 2 (*green asterix*). The zoom-in panel shows an interesting vascular detail on what seems to be the contour of the healthy myelum. (**B**) Bmode corresponding to the µDoppler-image in panel A. (**C**) Sagittal µDoppler-image, again spanning both hemangioblastoma foci. (**D**) Axial image of the most cranial (*yellow asterix)* hemangioblastoma. Sag, Sagittal; Ax, Axial.

**Figure 4:**
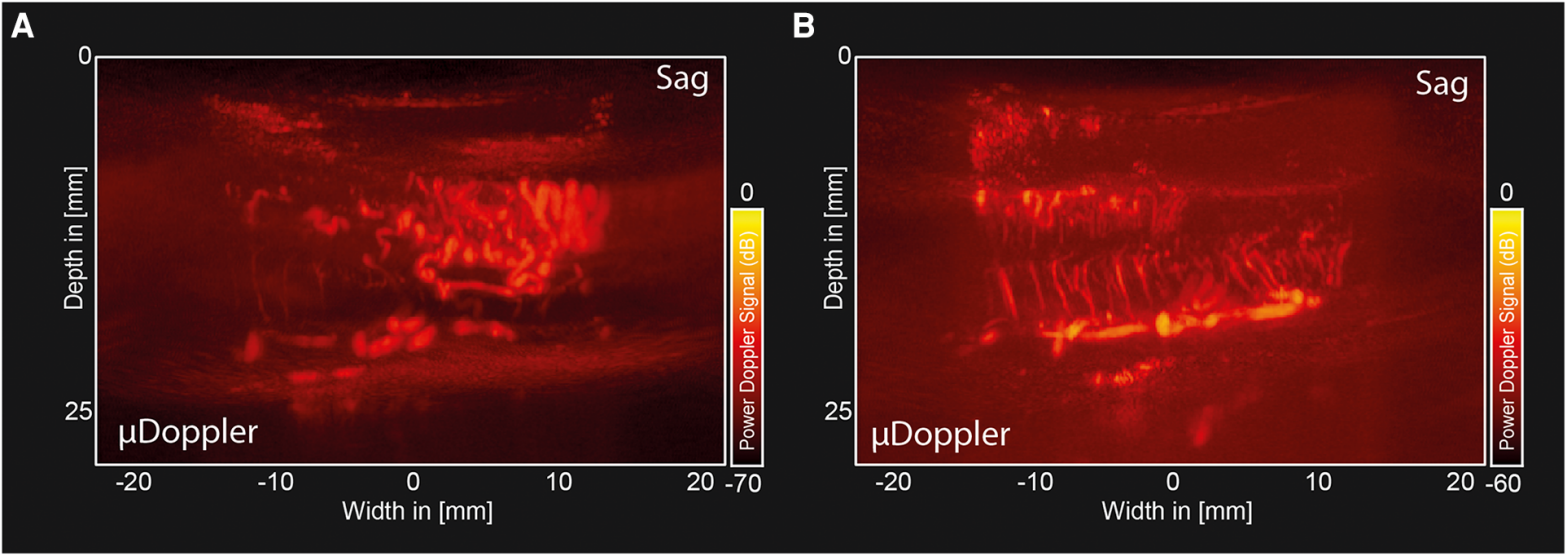
Pre-and post-resection µDoppler-images of a hemangioblastoma. (**A**) Sagittal µDoppler-images made pre-resection, showing a larger network of prominent vessels, similar to the ones seen pre-operatively in MRA. (**B**) Sagittal µDoppler-images made post-resection, showing the decompressed myelum.

2D-µDoppler was able to identify an intricate microvascular network inside both hemangioblastoma foci (Figures 3A–C) None of these details were visible in the pre-operative MRA (Figure 1E). Zooming in on one of the vascular details in the sagittal µDoppler-image (Figure 3A), we see the submillimeter level of detail µDoppler is able to provide in real-time during the surgery. Interestingly, when comparing the sagittal µDoppler-image (Figure 3A) to its conventional greyscale Bmode counterpart (Figure 3B), this particular vessel seems to demarcate the contour of the compressed healthy myelum. In Figure 3D we see an axial image of the most cranial (*yellow asterix)* hemangioblastoma, again revealing µDoppler's ability to detect microvascular details. Figure 4A shows a final pre-resection sagittal image of the spinal cord, now focusing on a larger network of more prominent vessels, seen directly caudal from lesion 1. These vessels seem to be similar to the ones seen pre-operatively in MRA (Figure 1E), where they were suspected to be formed due to compression and/or congestion.

### 4.2. Post-resection μDoppler images

Figure 4B shows a post-resection sagittal µDoppler-image of the decompressed humans spinal cord, showing key anatomical features such as the Ventral Spinal Artery (VSA) and peripheral branches from the pial plexus, penetrating the spinal cord.

### 4.3. Color Doppler images (CDIs)

**Figure 5:**
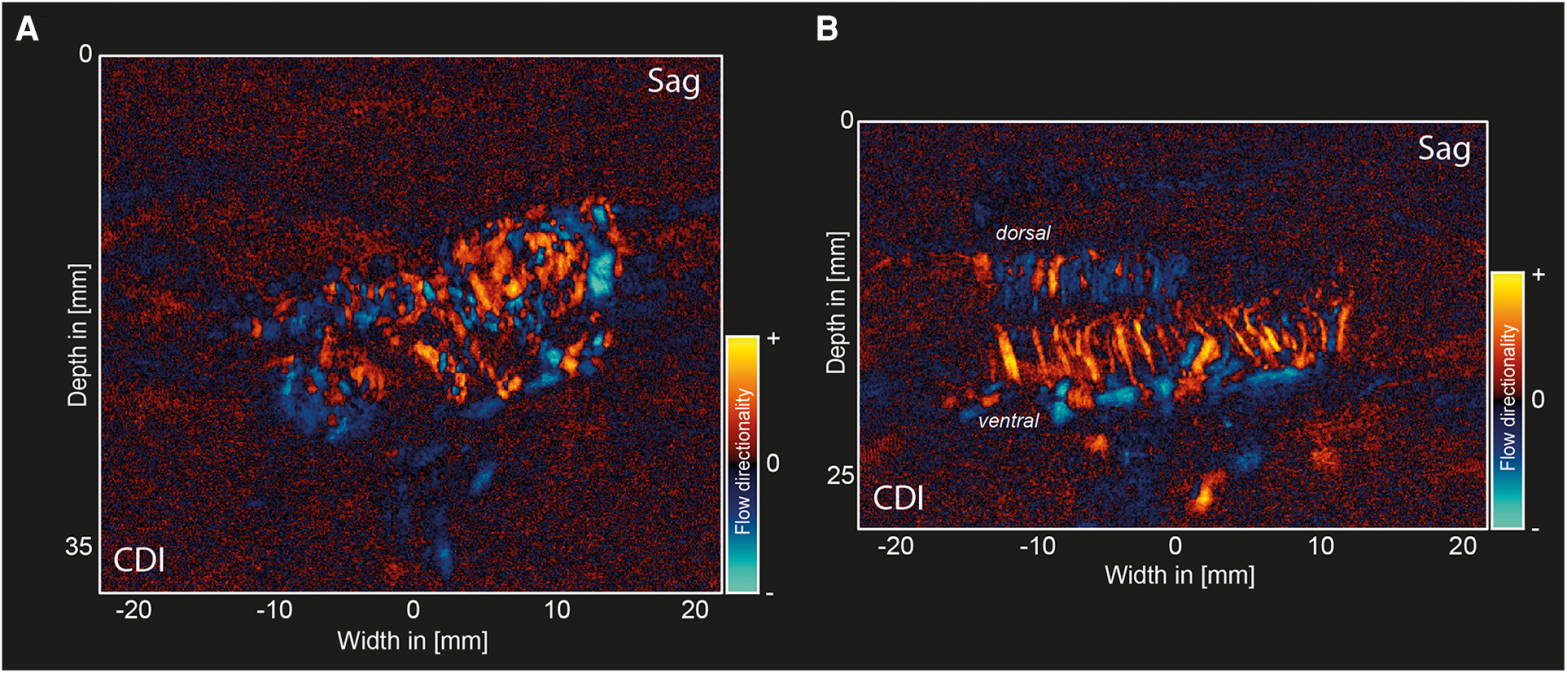
Color Doppler images (CDIs) pre-and post-resection of a hemangioblastoma. (**A**) Pre-resection, sagittal CDI of the same plane as in Figure 3A, The color axis depicts flow directionality with positive values indicating flow *towards* the transducer, and negative values indicating flow *away* from the transducer. (**B**) Sagittal CDI of the same plane previously shown in Figure 4B. CDI, Color Doppler Image.

Figure 5A shows the CDI of the same plane shown in Figure 3A, demonstrating the differences in flow directionality in the two hemangioblastoma foci. Figure 5B shows the CDI of the decompressed myelum post-resection of both foci (same plane as shown in Figure 4B). As we expect based on the anatomical organization of the spinal cord, the penetrating peripheral branches from the pial plexus clearly present with different flow directionalities in the dorsal and ventral side of the spinal cord.

### 4.4. Post-operative patient outcomes

Directly post-operatively, neurological examination showed similar motor scores for the right leg as were seen pre-operatively. The patient underwent an intensive rehabilitation programme and was seen for regular check-ups with MRI-scans every 6 months. One year after surgery, walking and standing had subjectively improved based on patient report, without significant change on the MRC-scale for both legs. The patient expressed to be satisfied with the surgical outcomes. The one-year MRI showed a slight growth of tissue in the thoracic surgical region, which now warrants more close monitoring with more regular MRI-scans (every 3 months).

## 5. Discussion

To the best of knowledge, this work presents the first µDoppler-images of human spinal hemangioblastomas. We show how µDoppler has the ability to detect intricate, intralesional microvasculature, which is otherwise not available pre- or intra-operatively with the currently available clinical techniques such MRI, MRA or conventional ultrasound. Having access to a real-time, high-resolution technique which can visualize hemodynamics in particular, could be valuable to support the neurosurgeon in their balancing act between removing too much or too little of the hemangioblastoma intra-operatively. The hope is that by having access to demarcating microvascular details as we show here (for example Figures 3A–C, Figure 4A), combined with µDoppler's real-time hemodynamic information such as flow directionality (Figure 5), neurosurgeons will be able to identify key anatomical features, and how these change as surgery progresses. Within the neurosurgical field, colleagues such as Siller et al. recommended to use vascular details to guide resection. For example, to first coagulate and transect feeding arteries before tumor resection and occlusion of the draining veins (12). Being able to identify these vessels easily and reliably, as well as monitor in real-time what would be the hemodynamic consequences of a surgical decision, would be an addition to the neurosurgeon's toolbox.

However, in this first description of intra-operative µDoppler-imaging applied to hemangioblastoma, we have not described any immediate surgical impact on the case. In fact, the Dutch medical-ethical committee explicitly restricted the use of our experimental technique for surgical decision-making at this point of the study. Until now, the resolution we could achieve while imaging the spinal cord with µDoppler was not available intra-operatively using ultrasound, with only CEUS coming somewhat close (20, 21, 26). Therefore, this current report aims to create scientific awareness of the availability and image quality of µDoppler, hoping to inspire others working on hemangioblastoma to join in studying its surgical potential.

What is more, in line with our previous report on µDoppler-imaging in the context of cerebral AVMs (32), real-time imaging of spinal cord hemodynamics and morphology has many other benefits than improving surgical decision-making alone: increasing our understanding of neurovascular pathology. Up until now, there is only a handful of reports in literature showing images of the human spinal cord (37–39). This means that, as we continue to acquire µDoppler-images of the spinal cord in both health and disease, a wealth of new information becomes available for study. This could for example improve our understanding of how hemangioblastomas and other spinal cord tumors manifest and grow. But also outside of oncology, fields such as neurotrauma and spinal cord injury in particular, would benefit from understanding physiological vascular patterns in the human spinal cord (36). Hopefully, this kind of knowledge could in turn circle back to improve surgical procedures and ultimately, patient outcomes.

To truly add to surgical decision-making in the future, we will need to take our limited 2D-images and move to real-time 3D-imaging in the OR, an effort currently being undertaken by our team and many others alike. For 3D to succeed, but also to improve 2D-image quality, we will need to find better ways to deal with the breathing motion artefacts. In this paper, we chose to avoid motion compensation altogether by selecting specific, relatively stable PDIs which we acquired using our intra-operative surgical arm. Although our approach with the surgical arm has minimized motion artefacts, the ideal scenario would be to correct or compensate for the breathing motion artefact altogether.

Motion correction would be especially essential in the context of functional mapping of the spinal cord. As discussed in the introduction, the microvascular hemodynamics measured with µDoppler-imaging form the basis of ‘functional Ultrasound’ (fUS) (30, 42). Through the process of neurovascular coupling (NVC), hemodynamics can serve as an indirect measure of neuronal activity and therefore brain functionality (30, 31, 43). So far, two teams have demonstrated how fUS can be used to map brain functionality during awake brain tumor resections, where patients were able to perform simple functional tasks such as lip pouting or word repetition (29, 31). Although spinal cord tumor resections are not performed awake, they are at times guided by neurophysiological signals or electrical stimulation during IONM, which can serve as functional task patterns to use for functional mapping of the spinal cord. In animals, fUS proved to be reliable in tracking of spinal cord responses to patterned epidural electrical stimulations (34). The authors also demonstrated how fUS had a higher sensitivity in monitoring spinal cord response than electromyography, with fUS being able to detect spinal cord signals subthreshold to motor response level of SCS (34). Similarly, a first application of fUS in the human spinal cord during standard-of-care implantation of a SCS paddle lead showed the technique's ability to capture functional response in the axial plane after electrical stimulation in the context of pain treatment (39). A future direction of our team will be to expand µDoppler-imaging to IONM-guided functional mapping of the spinal cord during spinal cord tumor resections. One important point of focus in this effort will be to increase our understanding of the similarities and differences between the brain and spinal cord in terms of NVC.

This case report marks the first application of µDoppler-imaging in the case of a patient with two thoracic spinal hemangioblastomas. We demonstrate how µDoppler is able to identify intra-operatively and with high-resolution, hemodynamic features of the lesion. In contrast to pre-operative MRA, µDoppler could identify intralesional vascular details in real-time during the surgical procedure, without the need for a contrast-agent. Additionally, our technique was able to capture highly detailed post-resection images of physiological human spinal cord anatomy. Although immediate surgical impact could not be achieved in this single case report, we hope this demonstration will add to scientific awareness of the availability of µDoppler-imaging, as well as the quality of its images when applied to new contexts such as hemangioblastoma.

## Data Availability

The raw data supporting the conclusions of this article will be made available by the authors, without undue reservation.
